# Memantine augmentation in clozapine-refractory schizophrenia: a randomized, double-blind, placebo-controlled crossover study

**DOI:** 10.1017/S0033291716000398

**Published:** 2016-04-06

**Authors:** S. R. T. Veerman, P. F. J. Schulte, J. D. Smith, L. de Haan

**Affiliations:** 1 Mental Health Service Organization North Holland North, Community Mental Health Division, Flexible Assertive Community Treatment, Alkmaar, The Netherlands; 2 Mental Health Service Organization North Holland North, Division for Specialized Treatment, Treatment Center for Bipolar Disorders, Alkmaar, The Netherlands; 3 Department of Psychiatry and Behavioral Sciences, Northwestern University, Feinberg School of Medicine, Center for Prevention Implementation Methodology, Chicago, IL, USA; 4 Early Psychosis Department, Academic Medical Center, University of Amsterdam, Academic Psychiatric Center, Arkin, Amsterdam, The Netherlands

**Keywords:** Antipsychotics, cognitive functioning, memantine, randomized controlled trials, therapy-resistant schizophrenia

## Abstract

**Background:**

Dysfunction of neuroplasticity due to *N*-methyl-d-aspartate (NMDA) receptor hypofunction may be a causal factor for memory and executive dysfunctioning in schizophrenia. Deregulation of NMDA transmission in the prefrontal cortex may also explain negative and positive symptoms. Clozapine augmentation with memantine targets altered NMDA receptor-mediated neurotransmission in schizophrenia and showed substantial beneficial effects on several symptom domains in a small proof-of-concept study. We evaluate effects of memantine add-on treatment to clozapine for memory and executive function, and negative and positive symptoms in schizophrenia.

**Method:**

Clozapine-treated patients with refractory schizophrenia were randomly assigned to 12 weeks of double-blind adjunctive treatment with memantine (*n* = 26) or placebo (*n* = 26). Crossover occurred after a 2-week placebo wash-out period. Primary endpoints were change from baseline to 12 weeks treatment and 14 weeks to 26 weeks treatment on memory and executive function using the Cambridge Neuropsychological Test Automated Battery (CANTAB), Positive and Negative Syndrome Scale (PANSS), and Clinical Global Impression Severity Scale (CGI-S). Side effects were assessed using the Liverpool University Neuroleptic Side-Effect Rating Scale.

**Results:**

When compared with placebo, memantine improved a composite memory score comprising verbal recognition memory and paired associates learning task scores on the CANTAB (effect size = 0.30) and PANSS negative subscale score (effect size = 0.29). Side effects were mild and transient.

**Conclusions:**

In patients with clozapine-treated refractory schizophrenia, memantine addition significantly improved verbal and visual memory and negative symptoms without serious adverse effects. These results justify further investigations on long-term memantine augmentation to clozapine in treatment-resistant schizophrenia.

## Introduction

Although clozapine is efficacious for treatment-resistant schizophrenia patients, as many as 70% of patients show only a partial response (Hasan *et al.* 2012). Polypharmacy is frequently used; however, evidence concerning additional pharmacological treatment of refractory schizophrenia is limited (Muscatello *et al.* 2014; Veerman *et al.* 2014*a*, *b*). Novel avenues of research are needed to bring about improved drug treatment of schizophrenia.

On the basis of the glutamate hypothesis, with hypofunction of the glutamate *N*-methyl-d-aspartate (NMDA) receptor as an underlying mechanism for schizophrenia (Stone *et al.* 2007; Kantrowitz & Javitt, 2010), glutamate modulators can be seen as promising antipsychotic agents (Veerman *et al.* 2014*c*). The glutamate hypothesis of schizophrenia stipulates that hypofunction of the NMDA receptor is responsible for excitotoxic neurodegeneration, dysfunction of neuroplasticity and dysregulation of downstream neurons in response to glutamate release, resulting in cognitive impairment and negative symptoms (Javitt & Zukin, 1991; Bressan & Pilowsky, 2000; Howes & Kapur, 2009; Orellana & Slachevsky, 2013). Positive symptoms may develop through disinhibition of prefrontal cortical γ-amino butyric acid (GABA) interneurons, which are responsible for recurrent inhibition of pyramidal neurons (Homayoun & Moghaddam, 2007).

Memantine acts as a low-affinity type, uncompetitive, non-selective and voltage-dependent NMDA receptor antagonist (Parsons & Gilling, 2007). Memantine is licensed for treatment of moderate-to-severe Alzheimer's disease (AD) (Areosa *et al.* 2005). Efficacy in patients with moderate-to-severe AD was demonstrated in a meta-analysis of six randomized placebo-controlled trials showing modest beneficial effects on global status and cognition after treatment with memantine (Winblad *et al.* 2007). Memantine has a favorable safety and tolerability profile (Farlow *et al.* 2008).

Favorable effects of memantine addition to non-clozapine antipsychotics described in case reports and open studies were replicated in only one of three placebo-controlled trials of memantine in combination with non-clozapine antipsychotics (Lieberman *et al.* 2009; Lee *et al.* 2012; Rezaei *et al.* 2013). However, memantine is thought to be more promising as an adjunctive therapy to clozapine than to non-clozapine antipsychotics. One small 12-week randomized, placebo-controlled trial (*n* = 21) demonstrated efficacy of memantine augmentation in patients with partial remission of negative symptoms of schizophrenia on clozapine treatment with large effect sizes (ESs) for overall symptoms (ES = 2.75), positive symptoms (ES = 1.38), negative symptoms (ES = 3.33) and global cognitive functioning (ES = −1.32) (de Lucena *et al.* 2009).

The favorable effects of memantine augmentation to clozapine may be related to their conjunct action on NMDA receptors. This particular combination modulates glutamatergic neurotransmission at multiple levels (Veerman *et al.* 2014*c*): clozapine induces both up-regulation of *α*-amino-3-hydroxy-5-methyl-4-isoxazolepropionic acid (AMPA) receptors and NMDA receptors (Yeun *et al.* 2010; Tanahashi *et al.* 2012), and memantine may enhance further up-regulation of NMDA receptors causing activation in the presence of a strong stimulus (Joshi *et al.* 2007). NMDA receptors are highly expressed in the hippocampus (Bliss & Collingridge, 1993). Improvement of hippocampal dysfunction and functional connectivity between brain circuits, involving the prefrontal cortex (PFC) through NMDA-receptor mediated neuroplasticity, explains why combination therapy of clozapine and memantine possibly targets two specific cognitive domains: impaired memory and executive function.

Inspired by the unique functional psychopharmacological characteristics of the memantine–clozapine combination and the substantial positive findings of the first proof-of-concept study, we conducted a second, larger and more elaborate trial studying effects of adjunctive memantine therapy on memory, executive function and symptom severity in clozapine-treated patients suffering from refractory schizophrenia.

## Method

### Study design

**Fig. 1.**
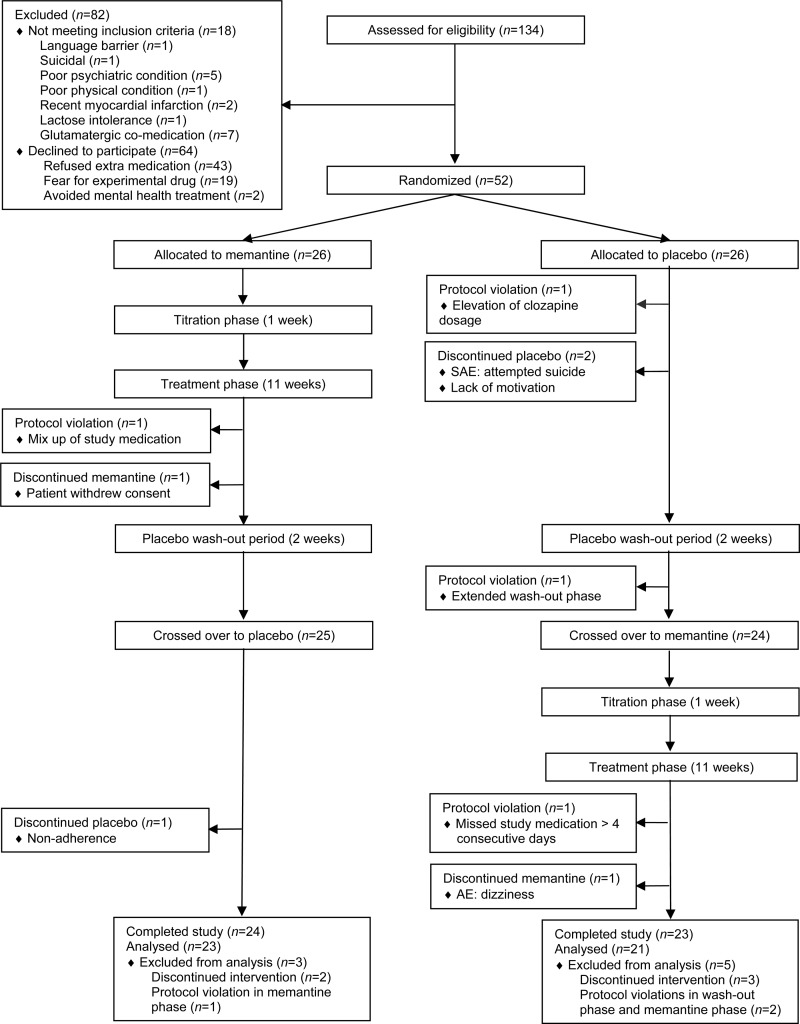
Patient disposition in a double-blind, randomized, placebo-controlled trial of memantine as adjunctive treatment to clozapine in refractory schizophrenia. SAE, Serious adverse event; AE, adverse event.

The study was approved by the Central Committee on Research Involving Human Subjects and the Medical Research Ethics Committee (MREC) of Alkmaar Medical Center and was conducted in accordance with the Declaration of Helsinki (World Medical Association, 2013). The study was a 26-week single-center, double-blind trial that was randomized and placebo controlled. The trial consisted of two crossover, 12-week treatment phases and a placebo wash-out period of 2 weeks in the 13th and 14th week to avoid carryover effects (Fig. 1). Clozapine dosage and use of concomitant medications were at the discretion of the treating psychiatrist and remained as much unaltered as possible throughout the study. Subjects were randomly assigned to receive an identical number of either memantine or placebo tablets. During the memantine phase a dosage of 10 mg taken once daily was built up after 1 week to 20 mg taken once daily during 11 weeks as add-on therapy to ongoing clozapine treatment. The dose of 20 mg/day was similar to the dosage used in all four randomized placebo-controlled trials in patients with schizophrenia (de Lucena *et al.* 2009; Lieberman *et al.* 2009; Lee *et al.* 2012; Rezaei *et al.* 2013).

Randomization to starting with either memantine or placebo was designated on a 1:1 basis in blocks of four. The allocation sequence was produced independently by the pharmacist of the VU Medical Center in Amsterdam. The code was concealed for patients, care providers, raters and investigators until all subjects had completed the trial and data had been entered into a computer data file.

### Study population, inclusion and exclusion criteria

The study was performed from August 2013 until August 2014, at 12 Flexible Assertive Community Treatment (FACT) facilities of the Mental Health Service Organization North Holland North (Netherlands) (van Veldhuizen, 2007). The original eligibility criterion of ‘out-patients’ was broadened to patients living either independently or in a sheltered home and patients admitted to open long-stay wards, receiving care from an out-patient facility. This was reported to the MREC of Alkmaar Medical Center. Eligible subjects were between the ages of 18 and 60 years, met Diagnostic and Statistical Manual of Mental Disorders, fourth edition (DSM-IV) criteria for schizophrenia on the Mini International Neuropsychiatric Interview Plus (MINI-Plus) (Overbeek *et al.* 1999), and failed to achieve remission criteria proposed by Andreasen *et al.* (2005), defined as simultaneous ratings of mild or less (⩽3 points) on eight of the following Positive and Negative Syndrome Scale (PANSS) items: P1 delusions, G9 unusual thought content, P3 hallucinatory behavior, P2 conceptual disorganization, G5 mannerisms and posturing, N1 blunted affect, N4 passive or apathetic social withdrawal, N6 lack of spontaneity and flow of conversation. At inclusion, duration of clozapine therapy was at least 6 months with a minimum of 12 weeks with a clozapine plasma level above 350 ng/ml or intolerability to achieve this threshold (Schulte, 2003). Patients with a recent deterioration needing treatment in an acute treatment ward were not included. Other exclusion criteria included pregnancy, lactating women, and female subjects without adequate contraception, known hypersensitivity to memantine, co-medication with glutamate modulators, lactose intolerance, uncontrolled epilepsy, myocardial infarction, uncontrolled hypertension, renal insufficiency, liver failure or AD (Wesemann *et al.* 1983). The sample size was calculated at 52, based on an ES of 0.55 (*α* = 0.05, power = 0.80) and accounting for an estimated discontinuation rate of 20%. After complete description of the study to the subjects, written informed consent was obtained by the principal investigator. Care providers distributed study medication and monitored compliance on a daily basis in patients in sheltered homes and open long-stay wards and on a weekly basis in out-patients.

### Clinical assessments

We used the Cambridge Neuropsychological Test Automated Battery (CANTAB), a computerized, non-linguistic cognitive testing battery (Levaux *et al.* 2007). Test selection was based on six cognitive domains, recommended by the Measurement and Treatment Research to Improve Cognition in Schizophrenia (MATRICS): reaction time and psychomotor speed, sustained visual attention, verbal memory, visuospatial memory, learning and association ability, working visuospatial memory and strategy use, spatial planning and motor control and emotion recognition (Nuechterlein *et al.* 2008; Barnett *et al.* 2010). One cognitive domain of the MATRICS Consenus Cognitive Battery (MCCB) was not assessed, because Intra/Extra-Dimensional Set-Shifting (IED) for reasoning and problem solving was too difficult for our patient population with severe cognitive disturbances.

Two cognitive domains were selected as primary outcomes: memory and executive function (see Table 2). Memory was assessed by computing a composite score of the sum of the CANTAB scores of four tasks: verbal recognition memory (VRM) free recall and VRM recognition, and paired associates learning (PAL) total errors and PAL first trial memory score. To reduce practice effects a parallel form of the VRM task, equivalent in difficulty, was used for the second and fourth measurement. Executive function was assessed by computing a composite of three CANTAB task scores: One Touch Stockings of Cambridge (OTS) problems solved on first choice, and spatial working memory (SWM) strategy and SWM between errors.

The PANSS was used to assess severity of positive, negative and total symptoms of schizophrenia (Kay *et al.* 1987). We assessed the effect of memantine on two subdomains of negative symptoms: (1) expressive deficits [flat affect (N1), poor rapport (N3), lack of spontaneity and flow of conversation (N6), mannerisms and posturing (G5), motor retardation (G7) and avolition (G13)]; and (2) social amotivation [emotional withdrawal (N2), passive/apathetic social withdrawal (N4) and active social avoidance (G16)] (Liemburg *et al.* 2013; Millan *et al.* 2014). Global severity of psychopathology was determined by using the Clinical Global Impression Severity Scale (CGI-S) (Guy, 1976).

Careful clinical procedures were performed to assess safety and tolerability of memantine add-on therapy to clozapine. Physical examination included measurements of waist circumference and blood pressure. Regular controls of white blood cell count and differentiation were combined with measurements of liver and renal function, blood glucose, lipids and plasma clozapine level (12 ± 0.5 h after ingestion). The occurrence and intensity of side effects were assessed by self-rating on the Liverpool University Neuroleptic Side-Effect Rating Scale (LUNSERS) (Day *et al.* 1995) augmented with rating of Likert scales for possible side effects of memantine (thrombosis, dyspnoea, and mycosis).

All outcomes were rated before treatment initiation, after 12 weeks, after 14 weeks, and after 26 weeks. Two raters were trained in diagnostic interviewing and all clinical assessments. Inter-rater exact and adjacent agreement (within one scale point) was 96% for the PANSS, based on seven assessments (two live patient interviews and five videotaped patient interviews).

Adverse events (AEs) were defined as any undesirable experience occurring to a subject during the study, whether or not they were considered to be related to memantine ingestion. All AEs, reported by either the subject or treatment staff, were recorded. Admission to a psychiatric hospital was no reason to break the code or for withdrawal from the study. A medical emergency was the only reason to break the code and withdraw the subject from the study.

### Statistical analysis

To determine the effects of memantine on the hypothesized cognitive functions, schizophrenia symptoms, safety measures, and side effects, the two phases (memantine or placebo) of the crossover trial were compared using a linear mixed-effects model conducted in SPSS Statistics version 22.0.0 (SPSS Inc., 2014). This analytic approach can estimate random and fixed effects simultaneously (Putt & Chinchilli, 1999). A natural log transformation was applied to cognitive function scores assessed via the CANTAB, which are non-normally distributed, so that estimates from the linear mixed-effects models would be trustworthy.

We conducted the analyses using an intention-to-treat (ITT) analytic approach and a per-protocol analytic approach, which included only protocol completers. Protocol completion was defined as having completed both treatment phases without a serious protocol violation in the memantine phase. There was no significant difference in study completion rate by random group assignment (group 1 = 23/26, group 2 = 21/26; χ^2^_1_ = 1.209, *p* = 0.47). We also tested a number of covariates in the model, which are potentially related to the dependent variables. Covariates included patient age and gender, years of education, age of onset, duration of psychosis and duration of untreated psychosis. We followed the backwards trimming method described by Singer & Willett (2003) to construct our models: as covariates were entered into the model, one at a time, those that were significantly related to one of the model's parameters were retained. Only the patient's years of education variable was related to the slope paramater; thus, this variable was retained in the final model and the others were discarded.

We first tested a model with random intercepts. However, the models failed to converge or produced errors in the Hessian matrix, so the intercept parameter was fixed to ensure trustworthy parameter estimates. The slope parameter was treated as a fixed effect after it was found that models with random slopes resulted in worse model fit. This indicates that models with fixed intercepts and slopes (i.e. constraining these parameters to be equal across participants) do not significantly differ from models in which these parameters are individually estimated. All tests of significance were two-tailed, and *α* was set to 0.05. Standardized ESs (Cohen's *d*) were calculated (Cohen, 1988). We performed a *post-hoc* analysis to assess whether memantine had a more pronounced effect on expressive deficits or social amotivation. To evaluate possible differences between in-patients admitted to long-stay wards and other included patients we performed a *post-hoc* analysis of demographic variables and baseline characteristics and also conducted a *post-hoc* analysis of out-patients, excluding patients admitted to long-stay wards.

### Reliability of analyses

To include the full, randomized sample in the analyses, restricted maximum likelihood estimation was used, which has been shown to provide unbiased estimates when data are missing at random or missing completely at random (MCAR) (Little & Rubin, 2002). There was some degree of missing data in our sample (see Tables 2 and 3 and online Supplementary Tables S1 and S2 for valid *n*'s), but the data were determined to be MCAR (39) (χ^2^_737_ = 144.40, *p* = 1.00), so the missing data did not introduce bias into the analyses.

Two steps were taken in the analytic strategy to address unique sources of potential bias in the crossover design: (1) a period effect parameter and a period × treatment interaction were tested to determine whether the order in which memantine was received was related to the outcome and whether there was a larger effect in one of the phases of the trial^2^; and (2) carryover effects were controlled for in all analyses.

Other possible confounders were analysed, such as change in clozapine dosage, concomittant medication, substance use, and use of psychotherapy during the study. The element of expectation of the placebo response was tested by examining whether pretrial expectations of positive benefits of memantine moderated the effect on the outcomes of active drug and placebo. Further, we analysed the degree of successful blinding by examining the degree of accurate appraisal of receipt of memantine from the perspective of the patient and the rater.

## Results

### Baseline characteristics

Fig. 1 presents participant screening and enrolment flow data. A total of 134 patients were screened, of whom 116 patients met inclusion criteria; 64 eligible patients refused participation. The remaining 52 patients gave informed consent and were enrolled. Table 1 presents demographic and clinical characteristics of the study population. Omnibus tests revealed that the two groups did not differ on any parameter except for significantly higher expectations for improvement in daily activities and living conditions in the group first assigned to placebo, compared with the group first assigned to memantine. A *post-hoc* analysis showed no significant differences between the six in-patients and the 46 out-patients on any of the demographic variables or baseline characteristics.

**Table 1.** Patient demographics and baseline characteristics

			Omnibus test	
Characteristic	*n* (%)	Mean (s.d.)	χ^2^/*F*	*p*
Gender			χ^2^_1_ = 0.923	0.52
Female	13 (25)			
Male	39 (75)			
Age, years		42.35 (9.55)	*F*_1,51_ = 3.165	0.08
Years of education		12.23 (1.79)	*F*_1,51_ = 0.596	0.44
Education level			χ^2^_1_ = 0.202	0.90
Low	28 (53.8)			
Middle	17 (32.7)			
High	28 (13.5)			
Living conditions				
Independently	30 (57.7)			
Sheltered home	16 (30.8)			
Long-stay department	6 (11.5)			
Age of onset, years		19.46 (4.68)	*F*_1,51_ = 2.848	0.10
Duration of untreated psychosis, months		35.04 (39.80)	*F*_1,51_ = 0.818	0.37
Duration of illness, years		22.88 (7.99)	*F*_1,51_ = 1.24	0.27
Alcohol use	18 (34.6)		χ^2^_1_ = 0.000	1.00
Cannabis use	9 (17.3)		χ^2^_1_ = 1.209	0.47
Cocaine use	1 (1.9)		χ^2^_1_ = 1.020	1.00
Amphetamine use	1 (1.9)		χ^2^_1_ = 1.020	1.00
Clozapine daily dose, mg		350.00 (182.84)	*F*_1,51_ = 0.171	0.68
Clozapine monotherapy (single antipsychotic)	36 (69.2)		χ^2^_1_ = 2.342	0.22
Combination clozapine + second antipsychotic^a^	16 (30.8)		χ^2^_1_ = 3.250	0.13
Combination clozapine + antidepressant	30 (57.7)		χ^2^_1_ = 0.315	0.78
Combination clozapine + mood stabilizer^b^	6 (11.5)		χ^2^_1_ = 3.014	0.19
Combination clozapine + benzodiazepine	18 (34.6)		χ^2^_1_ = 0.000	1.00
Psychotherapy during past 6 months	4 (7.7)		χ^2^_1_ = 0.000	1.00
Family members or partners involved in the study	26 (50.0)		χ^2^_1_ = 4.600	0.47
Expected improvement due to memantine		3.50 (0.918)	*F*_1,51_ = 0.818	0.37
None	2 (3.8)			
Very little	3 (5.8)			
Little	20 (38.5)			
Much	21 (40.4)			
Very much	6 (11.5)			
Expected improvement in personal hygiene		2.19 (1.37)	χ^2^_4_ = 4.671	0.46
Expected improvement in personal relationships		2.75 (1.17)	χ^2^_4_ = 3.444	0.49
Expected improvement in daily activities		3.00 (1.21)	χ^2^_4_ = 12.600	0.01
Expected improvement in living conditions		2.35 (1.27)	χ^2^_4_ = 11.200	0.04
Expected improvement in finances		1.83 (1.18)	χ^2^_4_ = 3.586	0.17

s.d., Standard deviation.

a Eleven patients received aripiprazole, two patients received quetiapine, one patient received olanzapine and one patient received zuclopenthixol acetate.

b Four patients received valproate and two patients received lithium.

### Clinical efficacy results

Analysis of effects using an ITT approach, presented in Table 2, indicated that all effects were in the direction of desired effect. The following primary outcome variables significantly improved during the memantine phase in comparison with the placebo phase: memory composite (*F*_4,655_, ES = 0.30, *p* = 0.032); PANSS negative symptoms (*F*_1,84_ = 4.170, ES = 0.29, *p* = 0.043). Other primary outcomes did not significantly improve after addition of memantine compared with placebo: executive function composite (*F*_1,84_ = 4.655, ES = 0.12, *p* = 0.395); PANSS positive symptoms (*F*_1,84_ = 1.008, ES = 0.15, *p* = 0.299); PANSS total symptoms (*F*_1,84_ = 1.869, ES = 0.19, *p* = 0.174); CGI (*F*_1,84_ = 0.591, ES = 0.11, *p* = 0.443). A *post-hoc* analysis on PANSS negative symptoms showed that memantine had a more pronounced effect on the expressive deficits subscale score (ES = 0.17) compared with the social amotivation subscale score (ES = 0.01). Table 2 contains the results of analyses on the individual CANTAB tasks comprising the two composite scores for reference only. No specific hypotheses were tested concerning the individual CANTAB tasks.

**Table 2.** Intention-to-treat analysis of primary treatment effects^a^

		Intention to treat (*n* = 52)			
Variable	CO	*n*	*F* test	ES	*p*
Cognitive domains and CANTAB tasks					
Verbal memory					
VRM free recall – total correct (phase 1)	0.00	40	11.818	0.48*	0.001
VRM recognition – total correct	0.09	40	1.421	0.17	0.235
Associative learning and short-term visuospatial memory					
PAL total errors (adjusted)	0.04	37	1.219	−0.15	0.271
PAL first trial memory score	0.09	37	5.778	0.34*	0.017
Memory composite	0.00	37	4.655	0.30*	0.032
Working visuospatial memory and strategy use					
SWM strategy	0.32	37	0.159	−0.06	0.690
SWM between errors	0.47	37	1.522	−0.17	0.219
Visual planning, reasoning and impulsivity					
OTS problems solved on first choice	0.02	39	0.048	0.03	0.827
Executive function composite	0.47	37	0.726	0.12	0.395
CGI-S	0.32	47	0.591	0.11	0.443
PANSS-P	0.02	44	1.088	0.15	0.299
PANSS-N	0.01	44	4.170	0.29*	0.043
PANSS total	0.00	44	1.869	0.19	0.174

CO, Carryover effect (*p* value of paired *t* test); ES, effect size (Cohen's *d*); CANTAB, Cambridge Neuropsychological Test Automated Battery; VRM, verbal recognition memory-immediate (free recall) and verbal recognition memory-delayed (recognition); PAL, paired associates learning; SWM, spatial working memory; OTS, One Touch Stockings of Cambridge; CGI-S, Clinical Global Impression Severity Scale; PANSS, Positive and Negative Syndrome Scale; PANSS-P, PANSS positive subscale; PANSS-N, PANSS negative subscale; PANSS total, PANSS total symptom score.

a All effects were in the direction of the desired effect.

* Significant beneficial effect.

This pattern of significant results in the ITT approach was consistent with the per-protocol analyses: memory composite (*F*_4,150_, ES = 0.31, *p* = 0.043); and PANSS negative symptoms (*F*_1,72_ = 3.514, ES = 0.21, *p* = 0.043). Tables 2 and 3 and online Supplementary Tables S1 and S2 summarize results and statistics of primary outcomes, side effects and safety measures.

**Table 3.** Side effects and safety measures

							Omnibus test	
Variable	Baseline (*n* = 52)	Memantine 1 (*n* = 25)	Placebo 1 (*n* = 24)	After wash-out (*n* = 44)	Memantine 2 (*n* = 21)	Placebo 2 (*n* = 24)	χ^2^/*F*	*p*
Memantine-related AE mycosis, *n* (%)							χ^2^_4_ = 5.750	0.33
None	46 (67.3)	23 (92)	20 (83.3)	35 (81.4)	12 (57.1)	22 (88.5)		
Very little	2 (3.8)	0 (0.0)	3 (12.5)	3 (7.0)	6 (28.6)	1 (4.2)		
Little	4 (7.7)	2 (7.7)	1 (4.2)	4 (9.3)	2 (9.5)	1 (4.2)		
Much	0 (0.0)	0 (0.0)	0 (0.0)	1 (2.3)	0 (0.0)	0 (0.0)		
Very much	0 (0.0)	0 (0.0)	0 (0.0)	0 (0.0)	1 (4.8)	0 (0.0)		
Memantine-related AE dyspnea, *n* (%)							χ^2^_5_ = 5.329	0.26
None	26 (50.0)	9 (36.0)	15 (62.5)	19 (43.2)	11 (52.4)	9 (37.5)		
Very little	6 (11.5)	10 (16.0)	5 (20.8)	8 (18.2)	5 (23.8)	4 (16.7)		
Little	13 (25.0)	4 (40.0)	3 (12.5)	14 (31.8)	4 (19.0)	8 (33.3)		
Much	6 (11.5)	0 (0.0)	1 (4.2)	0 (0.0)	0 (0.0)	3 (12.5)		
Very much	1 (1.9)	2 (8.0)	0 (0.0)	2 (4.5)	1 (4.8)	0 (0.0)		
Mean LUNSERS scores (s.d.)^a^								
Extrapyramidal symptoms	13.37 (4.06)	13.08 (4.18)	13.33 (5.31)	12.34 (3.94)	13.00 (4.30)	12.46 (3.12)	*F*_1,182_ = 1.598	0.21
Anticholinergic side effects	9.87 (3.46)	9.13 (3.66)	9.92 (3.67)	9.26 (3.04)	9.80 (3.86)	8.33 (2.79)	*F*_1,185_ = 1.323	0.25
Other autonomic side effects	9.27 (3.16)	8.42 (3.28)	9.33 (3.69)	8.95 (2.85)	9.11 (3.04)	8.37 (2.50)	*F*_1,184_ = 0.319	0.57
Allergic reactions	5.57 (1.66)	5.17 (1.83)	6.26 (2.49)	6.58 (2.99)	6.70 (3.01)	591 (2.52)	*F*_1,180_ = 4.839	0.03
Psychic side effects	23.63 (6.34)	22.36 (7.40)	24.58 (6.90)	22.44 (7.42)	22.75 (7.00)	22.08 (7.11)	*F*_1,183_ = 1.295	0.26
Hormonal side effects	8.20 (3.03)	7.96 (2.22)	8.33 (2.22)	8.54 (2.68)	8.72 (3.12)	7.38 (2.27)	*F*_1,178_ = 0.066	0.80
Miscellaneous side effects	6.43 (1.95)	6.54 (2.19)	6.96 (1.76)	6.86 (2.04)	7.37 (1.80)	6.83 (2.10)	*F*_1,184_ = 1.694	0.20
Red herrings	14.52 (4.72)	14.83 (4.35)	14.92 (4.49)	15.14 (4.94)	15.65 (5.21)	14.78 (4.73)	*F*_1,185_ = 0.801	0.37
Red herrings ⩾20	0.13 (0.35)	0.17 (0.38)	0.21 (4.95)	0.21 (0.41)	0.20 (0.41)	0.17 (0.39)	*F*_1,184_ = 0.446	0.51
Total	92.80 (21.061)	90.9 (23.95)	95.74 (0.12)	93.63 (23.27)	96.00 (27.27)	87.50 (21.94)	*F*_1,184_ = 0.041	0.84
Total without red herring items	78.41 (17.12)	75.95 (20.16)	80.70 (20.47)	78.69 (19.37)	80.43 (21.96)	72.85 (17.66)	*F*_1,161_ = 0.447	0.73
Laboratory measurement								
Mean clozapine plasma level, ng/ml (s.d.)	421.27 (224.61)	515.16 (240.43)	376.48 (185.11)	461.71 (219.61)	403.06 (179.90)	436.14 (157.07)	*F*_1,178_ = 1.387	0.24
Range, ng/ml	50–878	101–1060	52–671	60–1080	135–682	200–715		
Physical examination								
Mean waist circumference, cm (s.d.)	110.04 (13.82)	108.32 (14.71)	104.04 (11.72)	105.50 (13.89)	104.41 (12.16)	107.88 (14.86)	*F*_1,181_ = 1.205	0.27
Mean systolic blood pressure, mmHg (s.d.)	134.57 (18.77)	169.00 (170.28)	130.26 (12.59)	130.48 (15.94)	125.68 (12.82)	130.75 (11.84)	*F*_1,188_ = 1.954	0.16
Mean diastolic blood pressure, mmHg (s.d.)	88.00 (9.77)	86.72 (10.57)	87.04 (8.10)	85.95 (9.41)	86.14 (9.70)	83.88 (7.60)	*F*_1,188_ = 1.084	0.30

AE, Adverse event; LUNSERS, Liverpool University Neuroleptic Side-Effect Rating Scale; s.d., standard deviation.

^a^ Extrapyramidal symptoms: items 19, 29, 34, 37, 40, 43, 48; anticholinergic side effects: items 6, 10, 32, 38, 51; other autonomic side effects: items 15, 16, 20, 27, 36; allergic reactions: items 1, 35, 47, 49; psychic side effects: items 2, 4, 9, 14, 18, 21, 23, 26, 31, 41; hormonal side effects: items 7, 13, 17, 24, 46, 50; miscellaneous side effects: items 5, 12, 39, 44; red herrings: items 3, 8, 11, 12, 25, 28, 30, 33, 42, 45.

A *post-hoc* analysis showed no evidence to suggest that the six patients admitted to long-stay wards responded differently to memantine than the 46 out-patients. In comparison with the full sample of 52 patients, the ESs were nearly identical in magnitude (data available on request).

### Possible confounders

Tests for period effects and period × treatment interaction were non-significant across all outcomes. When matched-pair *t* tests were used to compare scores from the first baseline to the second baseline of the trial, carryover effects were significant for the memory composite from the CANTAB, PANSS positive, negative, and total scores. *t* Tests and a repeated-measures analysis of variance indicated no significant differences in clozapine levels across the four assessment times in the trial, suggesting that memantine has no effect on clozapine levels. Clozapine dosage (mean 350 mg, range = 75–1000 mg) remained unaltered in all subjects except in one subject in the placebo phase whose clozapine dosage was increased with 175 mg because of agitation and verbal aggression. Alterations in concomitant medications throughout the study were limited to changes in benzodiazepines in one subject in the placebo phase. Use of substances or psychotherapy did not significantly change during the trial (see online Supplementary Table S3). Tests of moderation based on low and high levels of expectation for improvement prior to beginning the trial revealed that the element of expectation did not contribute to the placebo effect. Blinding was successful, in that 45.7% of patients guessed correctly which group they had been randomized to. The raters were correct in 21.7% of cases.

### Safety and tolerability

No significant changes in liver and renal function were observed. Compared with placebo, memantine did not significantly affect metabolic parameters, such as waist circumference, blood pressure, blood glucose or lipids (see Table 3). The only significant increase in reported side effects while taking memantine was found on the Allergic Reactions subscale of the LUNSERS (rash, sensitivity to sun, unusual skin marks, and itchy skin).

Of all AEs (see Table 4), one report of dizziness, a common side effect of memantine in elderly patients with AD, was probably related to memantine. Complaints of dizziness were alleviated within 5 days of discontinuation of study medication. There were two reports of temporary increase in constipation, which were rated as possibly related to memantine. Both participants had been already treated with laxatives because of clozapine-induced constipation. We observed one serious AE (a suicide attempt during the placebo phase).

**Table 4.** Adverse events (n)

Adverse event	Memantine	Wash-out phase	Placebo
No adverse event	40	47	39
Dizziness	1		
Nausea and vomiting			1
Constipation	2		1
Flu-like symptoms		1	1
Toxic clozapine plasma level with flu-like symptoms		1	
Toxic clozapine plasma level without symptoms			1
Bronchitis	1		
Pneumonia and urinary tract infection	1		
Cardiac pain			1
Hip pain			1
Delirium	1		
Somnolence		1	
Anxiety	2	1	3
Increase in auditory hallucinations	2		
Agitation and verbal aggression			1
Admission to a psychiatric unit	2	1	2
Attempted suicide			1

## Discussion and conclusions

Inspired by the unique psychopharmacological characteristics of the memantine–clozapine combination and the substantial positive findings of the first proof-of-concept study we conducted a second proof-of-concept study with a larger sample size and a computerized cognitive test battery to ensure accurate and objective study data with minimized inter-rater variability to evaluate the efficacy of memantine as an adjunct to clozapine in refractory schizophrenia.

Memantine treatment added to clozapine was associated with significant improvement in memory (ES = 0.30). Memory-enhancing effects of the combination therapy of clozapine and memantine may be a result of up-regulation of synaptic NMDA receptor currents in the hippocampus, facilitating induction of long-term potentiation and therefore learning and memory (Kornmeier & Sosic-Vasic, 2012).

Memantine did not significantly improve executive function (ES = 0.12). Executive function is a central cognitive process, involving the PFC, corticocortical and corticosubcortal networks (Evans *et al.* 1997; Lesh *et al.* 2011). Enhancement of executive function would require effects on more elaborate networks and several cognitive domains including planning, working memory, strategy use, cognitive flexibility and ability to suppress impulsivity. Apparently, memantine addition does not improve the functioning in these networks, or alternatively longer treatment duration is needed.

Negative symptoms significantly improved with a small ES (ES = 0.29). Memantine affected diminished expression to a larger extent than social amotivation. Improvement of expressive deficits may be a result of increased signal transmission with an enhanced signal-to-noise ratio in the PFC (Geerts & Grossberg, 2006; Hasan *et al*. 2013) due to the particular combination of clozapine and memantine.

Memantine and placebo did not differ significantly with respect to adverse effects, except for mild and transient allergic symptoms.

Improvement of cognitive disturbances and negative symptoms is an important goal for treatment-resistant schizophrenia. Together these symptoms have a more pronounced impact on psychosocial functioning and quality of life than positive symptoms (Ventura *et al.* 2015). Clinical impairment of memory is one of the major disabilities in schizophrenia. Specifically, verbal memory is a strong predictor of functional outcome (Green, 1996). Favorable effects of memantine in combination with clozapine may be based on the neuroprotective properties and pharmacodynamic activities of this combination. In a recent proton magnetic resonance spectroscopy study anterior cingulate cortex glutamate levels were elevated in patients with treatment-resistant schizophrenia compared with patients with treatment-responsive schizophrenia, endorsing our hypothesis that memantine is specifically efficacious in refractory schizophrenia (Mouchlianitis *et al.* 2015).

The effect of memantine augmentation that we found is in line with the improvement of negative symptoms that has been found in five trials of addition of a glutamate antagonist to clozapine in partially responding schizophrenia patients (Goff *et al.* 2007 (study 926); Zoccali *et al.* 2007; Afshar *et al.* 2008; de Lucena *et al.* 2009; Muscatello *et al.* 2010). Topiramate and lamotrigine both showed favorable effects on negative symptoms in each of two trials, with ESs varying from 0.76 to 1.37 and 0.66 to 1.21, respectively. Cognitive functioning had been assessed with different cognitive test batteries in five double-blind, placebo-controlled randomized trials of clozapine augmentation with glutamate antagonists. In one trial of topiramate (Muscatello *et al.* 2010) and two trials of lamotrigine add-on therapy to clozapine (Goff *et al.* 2007 (study 926); Vayısoğlu *et al.* 2013), cognitive functions did not significantly change compared with placebo. However, two trials showed favorable results on cognition (Zoccali *et al.* 2007; de Lucena *et al.* 2009). In the study by Zoccali *et al.* (2007), the only cognitive function that significantly improved was semantic fluency after 24 weeks of addition of lamotrigine 200 mg daily.

The differences and similarities between our results and that of the 12-week memantine add-on study by de Lucena *et al.* (2009) are striking. De Lucena *et al.* (2009) found exceptionally large ESs on all treatment outcome parameters. Most striking was the large ES of 3.33 concerning negative symptoms (de Lucena *et al.* 2009). But also the effect on global cognitive functioning (ES = *−*1.32), as measured by the Mini-Mental State Examination (MMSE) (Folstein *et al.* 1975), was substantial. Caution is necessary, for efficacy of memantine was perhaps overestimated partly by chance in this relatively small sample study (21 patients) (Sinclair & Adams, 2014; Tajika *et al.* 2015). The crossover design of our larger double-blind randomized clinical trial eliminated the influence of between-subject variability on effect. The MMSE, used by de Lucena *et al.* (2009), does not measure executive functioning and detects cognitive deficits only at an advanced stage, because tasks for language and memory functions are extremely simple (Feher *et al.* 1992). We used a more sensitive and comprehensive cognitive test battery developed for assessment of cognitive impairments in schizophrenia intervention studies (Levaux *et al.* 2007). The Brief Psychiatric Rating Scale (BPRS), used by de Lucena *et al.* (2009), merely covers three negative symptom items (blunted affect, emotional withdrawal and psychomotor retardation). We used the more sensitive PANSS scale with seven items on the negative symptom subscale (Eckert *et al.* 1996) and four additional items on the general symptom subscale in the *post-hoc* analysis (Liemburg *et al.* 2013). Our patient population differed compared with that of the first memantine augmentation to clozapine study in mean age (42.35 *v*. 34.67 years) and mean duration of illness (22.88 years *v*. 17.84 years), CGI-S scores (6.15 *v*. 5.34) and mean total PANSS and total BPRS scores (81.21 and 14.38, respectively), suggesting that our patient population was more severely ill than were patients in the study by de Lucena *et al.* (2009). Although in our patients the severity of residual negative symptoms was comparable with the severity of persistent positive symptoms (mean PANSS negative subscale = 22.12, s.d. = 5.86; mean PANSS positive subscale = 21.02, s.d. = 6.34), negative symptoms prevailed in the study by de Lucena *et al.* (2009). While mean total BPRS score (14.38) corresponds with ‘markedly ill’ according to CGI-S (score 5) in the de Lucena study, our patient population was rated as severely ill (mean CGI-S score 6.15) due to prominent cognitive impairment. These differences between studies may partly explain the more moderate beneficial effects of memantine addition in our study. However, the difference in ES for all symptom domains between the de Lucena study and ours is very large. It has been demonstrated that among randomized trials, initially stronger effects are not unusual (Ioannidis, 2005; Tajika *et al.* 2015). Memantine was generally well tolerated in both studies. Although there were no drop-outs in the first study, one participant discontinued in our trial because of dizziness in the memantine phase.

The results of our study are limited by the short memantine treatment duration of 12 weeks. A longer treatment duration may result in more pronounced treatment effects associated with an improved glutamatergic balance, as was found in patients with AD. In a meta-analysis of six randomized, placebo-controlled trials of memantine treatment in 2311 patients with AD, symptoms of delusion were more improved after 24 to 28 weeks compared with 12 weeks (Puangthong & Hsiung, 2009). Furthermore, the crossover design resulted in carryover effects on several measures, including verbal memory and all PANSS subscales, despite a 2-week placebo wash-out period. Although the model controls for carryover effects, these cannot be discarded completely. Practice effects were minimized due to the fact that the number of subjects randomized to the placebo and memantine groups before crossover was the same and a parallel form was used for verbal memory. Tests on executive function, depending on strategy, show strong practice effects and low test–retest reliability (Lowe & Rabbitt, 1998). However, there is no research on practice effects using the CANTAB in patients with severe cognitive disturbances suffering from refractory schizophrenia. Although our study included more patients than the first investigation by de Lucena *et al.* (2009) our sample size is still relatively small (Sinclair & Adams, 2014). The results of our study need to be validated in a randomized multicenter long-term treatment study with a large sample size and enough power to clearly show a reduction of at least 25% of the baseline score in order to help further evaluate pro-cognitive properties of memantine in combination with clozapine in refractory patients and its potential to reduce negative symptoms associated with schizophrenia (Leucht *et al.* 2009).

In conclusion, we found evidence that addition of memantine may be a well-tolerated treatment option for cognitive impairments and negative symptoms in patients with clozapine-refractory schizophrenia, deserving further study.

## Notes

2 A period effect was included as a control only as the study does not have sufficient power to test for such an effect directly. Further, a period effect was not hypothesized.

## Supplementary material

For supplementary material accompanying this paper visit http://dx.doi.org/10.1017/S0033291716000398. click here to view supplementary material
