# Supplementary material for: Memantine augmentation in clozapine-refractory schizophrenia: a randomized, double-blind, placebo-controlled crossover study
Source: Psychol Med. 2016 Apr 6;46(9):1909–21. doi: 10.1017/S0033291716000398 (PMC4954262; doi:10.1017/S0033291716000398)
Supplement: Supplementary file 1 [file S0033291716000398sup001.zip › S0022112016003098sup001/S0033291716000398sup001.doc]

**Supplementary Table S1.** *Per protocol analysis of primary treatment effects*

|  | Per Protocol | | | |
| --- | --- | --- | --- | --- |
|  | (N=44) | | | |
| **Variable** | N | F | ES | P |
| Cognitive domains and CANTAB tasks |  |  |  |  |
| Verbal memory |  |  |  |  |
| VRM free recall - total correct (phase 1) | 38 | 13.332 | 0.55* | .000 |
| VRM recognition - total correct | 38 | 0.974 | 0.15 | .335 |
| Associative learning and short term visuo-spatial memory |  |  |  |  |
| PAL total errors (adjusted) | 35 | 0.871 | –0.14 | .352 |
| PAL first trial memory score | 35 | 7.331 | 0.41* | .008 |
| Memory Composite | 35 | 4.150 | 0.31* | .043 |
| Working visuo-spatial memory and strategy use |  |  |  |  |
| SWM strategy | 35 | 0.015 | –0.02 | .903 |
| SWM between errors | 35 | 0.154 | –0.06 | .696 |
| Visual planning, reasoning and impulsivity |  |  |  |  |
| OTS problems solved on first choice | 37 | 0.002 | 0.01 | .967 |
| Executive function Composite | 35 | 0.011 | 0.02 | .915 |
| CGI-S | 42 | 0.000 | 0.00 | .991 |
| PANSS-P | 41 | 0.718 | 0.09 | .398 |
| PANSS-N | 41 | 3.514 | 0.21* | .043 |
| PANSS total | 41 | 1.381 | 0.18 | .242 |
| N = number, F = F-test; ES = effect size (Cohen’s *d*); P = *p*-value; CANTAB = Cambridge Neuropsychological Test Automated Battery; VRM = verbal recognition memory-immediate (free recall) and verbal recognition memory-delayed (recognition); PAL = paired associates learning; OTS = One Touch Stockings of Cambridge; SWM = spatial working memory; CGI-S = Clinical Global Impression Severity Scale; PANSS = Positive and Negative Syndrome Scale; PANSS-P = PANSS positive subscale; PANSS-N = PANSS negative subscale; PANSS total = PANSS total symptom score.  All effects were in the direction of desired effect.  * Significant beneficial effect. | | | | |
