# Supplementary material for: Memantine augmentation in clozapine-refractory schizophrenia: a randomized, double-blind, placebo-controlled crossover study
Source: Psychol Med. 2016 Apr 6;46(9):1909–21. doi: 10.1017/S0033291716000398 (PMC4954262; doi:10.1017/S0033291716000398)
Supplement: Supplementary file 1 [file S0033291716000398sup001.zip › S0022112016003098sup001/S0033291716000398sup002.doc]

***Supplementary Table S2.*** *Results of primary treatment effects*

|  | Memantine 0 | | Placebo 0 | | | Memantine 1 | | | Placebo 1 | | | After wash-out | | | Memantine 2 | | | Placebo 2 | |
| --- | --- | --- | --- | --- | --- | --- | --- | --- | --- | --- | --- | --- | --- | --- | --- | --- | --- | --- | --- |
|  | (N=26) | | (N=26) | | | (N=25) | | | (N=24) | | | (N=44) | | | (N=21) | | | (N=24) | |
| **Variable** | Mean | SD | Mean | SD | Mean | | SD | Mean | | SD | Mean | | SD | Mean | | SD | Mean | | SD |
| Primary Parameter Cognitive Functioning (CANTAB) | | | | | | | | | | | | | | | | | | | |
| Verbal memory | | | | | | | | | | | | | | | | | | | |
| VRM free recall - total correct (phase 1) | 3.39 | 1.67 | 3.81 | 1.39 | 3.74 | | 1.88 | 4.32 | | 1.94 | 4.73 | | 1.82 | 4.76 | | 2.00 | 4.43 | | 1.89 |
| VRM recognition - total correct | 19.78 | 2.45 | 19.58 | 4.00 | 21.05 | | 2.46 | 20.09 | | 3.70 | 21.05 | | 2.41 | 20.43 | | 5.05 | 20.62 | | 1.63 |
| Associative learning and short term visuo-spatial memory | | | | | | | | | | | | | | | | | | | |
| PAL total errors (adjusted) | 74.14 | 45.96 | 64.92 | 45.70 | 73.83 | | 51.40 | 56.00 | | 45.61 | 56.63 | | 41.05 | 60.95 | | 44.33 | 73.57 | | 45.64 |
| PAL first trial memory score | 9.68 | 6.77 | 10.62 | 6.42 | 9.33 | | 7.00 | 10.05 | | 4.98 | 11.74 | | 6.15 | 10.55 | | 5.82 | 9.24 | | 6.39 |
| Memory Composite | | | | | | | | | | | | | | | | | | | |
|  | –38.48 | 53.12 | –30.92 | 53.44 | –36.32 | | 59.83 | –19.45 | | 50.66 | –15.83 | | 47.90 | –22.81 | | 49.92 | –39.29 | | 52.60 |
| Working visuo-spatial memory and strategy use | | | | | | | | | | | | | | | | | | | |
| SWM strategy | 19.50 | 3.29 | 16.08 | 4.69 | 18.35 | | 3.02 | 17.33 | | 3.51 | 18.11 | | 3.52 | 17.20 | | 4.20 | 17.63 | | 4.09 |
| SWM between errors | 25.55 | 11.17 | 19.62 | 11.69 | 22.00 | | 9.48 | 18.10 | | 12.31 | 19.05 | | 12.80 | 19.45 | | 9.85 | 22.74 | | 11.49 |
| Visual planning, reasoning and impulsivity | | | | | | | | | | | | | | | | | | | |
| OTS problems solved on first choice | 7.55 | 3.70 | 7.12 | 4.47 | 7.17 | | 3.07 | 7.52 | | 3.83 | 7.05 | | 3.80 | 7.50 | | 3.94 | 7.38 | | 2.85 |
| Executive funtion Composite | | | | | | | | | | | | | | | | | | | |
|  | 37.50 | 16.13 | –29.58 | 18.11 | –30.94 | | 15.92 | –27.90 | | 17.87 | –27.33 | | 19.25 | –29.15 | | 15.47 | –29.14 | | 19.89 |
| Other Primary Parameters | | | | | | | | | | | | | | | | | | | |
| CGI-S | 6.15 | 0.68 | 6.15 | 0.78 | 6.20 | | 0.71 | 6.08 | | 0.83 | 6.13 | | 0.77 | 6.09 | | 0.79 | 6.12 | | 0.68 |
| PANSS-P | 20.23 | 7.20 | 21.81 | 5.37 | 18.48 | | 6.21 | 18.71 | | 5.47 | 19.52 | | 5.82 | 19.05 | | 6.07 | 17.92 | | 5.85 |
| PANSS-N | 22.04 | 6.25 | 22.19 | 5.57 | 20.16 | | 6.57 | 20.08 | | 5.87 | 21.41 | | 6.17 | 20.73 | | 6.08 | 19.00 | | 6.43 |
| PANSS total | 80.04 | 18.02 | 82.38 | 15.59 | 74.36 | | 17.47 | 74.38 | | 18.07 | 77.11 | | 17.28 | 74.82 | | 17.74 | 70.96 | | 15.64 |
| SD = standard deviation; CANTAB = Cambridge Neuropsychological Test Automated Battery; VRM = verbal recognition memory-immediate (free recall) and verbal recognition memory-delayed (recognition); PAL = paired associates learning; OTS = One Touch Stockings of Cambridge; SWM = spatial working memory; CGI-S = Clinical Global Impression Severity Scale; PANSS = Positive and Negative Syndrome Scale; PANSS-P = PANSS positive subscale; PANSS-N = PANSS negative subscale; PANSS total = PANSS total symptom score. | | | | | | | | | | | | | | | | | | | |
