# Supplementary material for: Memantine augmentation in clozapine-refractory schizophrenia: a randomized, double-blind, placebo-controlled crossover study
Source: Psychol Med. 2016 Apr 6;46(9):1909–21. doi: 10.1017/S0033291716000398 (PMC4954262; doi:10.1017/S0033291716000398)
Supplement: Supplementary file 1 [file S0033291716000398sup001.zip › S0022112016003098sup001/S0033291716000398sup003.doc]

**Supplementary Table S3.** *Treatment expectations, experienced improvement, substance use, and psychotherapy*

|  | Baseline | | Evaluation | | Omnibus Test | | |
| --- | --- | --- | --- | --- | --- | --- | --- |
| **Variable** | Mean | SD | Mean | SD | Χ^2^ / B / *F* | β | *p* |
| Expected improvement in personal hygiene | 2.19 | 1.37 |  |  | Χ^2^*(df* = 4) = 4.671 |  | .46 |
| Expected improvement in personal relationships | 2.75 | 1.17 |  |  | Χ^2^*(df* = 4) = 3.444 |  | .49 |
| Expected improvement in daily activities | 3.00 | 1.21 |  |  | Χ^2^*(df* = 4) = 12.600 |  | .01 |
| Expected improvement in living conditions | 2.35 | 1.33 |  |  | Χ^2^*(df* = 4) = 11.200 |  | .04 |
| Expected improvement in finances | 1.83 | 1.18 |  |  | Χ^2^*(df* = 4) = 3.586 |  | .17 |
| Experienced improvement in personal hygiene |  |  | 1.65 | 1.08 | B = .57 (*SE =* .32) | .27 | .08 |
| Experienced improvement in personal relationships |  |  | 1.78 | 1.19 | B = .66 (*SE =* .33) | .28 | .05 |
| Experienced improvement in daily activities |  |  | 1.87 | 1.05 | B = .45 (*SE =* .32) | .22 | .17 |
| Experienced improvement in living conditions |  |  | 1.46 | 1.01 | B = .26 (*SE =* .32) | .13 | .42 |
| Experienced improvement in finances |  |  | 1.35 | .71 | B = .42 (*SE =* .21) | .30 | .05 |
|  | N | % | N | % |  |  |  |
| Alcohol use | 18 | 34.6 | 18 | 39.1 | *F*(1, 45) = .328 |  | .57 |
| Cannabis use | 9 | 17.3 | 7 | 13.5 | ^a^ |  |  |
| Cocaine use | 1 | 1.9 | 1 | 1.9 | ^a^ |  |  |
| Amphetamine use | 1 | 1.9 | 1 | 1.9 | ^a^ |  |  |
| Psychotherapy during trial | 4 | 7.7 | 6 | 13.0 | *F*(1, 45) = 2.731 |  | .11 |
| ^a^ Cannot be computed as there was no change in substance use status from baseline to evaluation for available data. Omnibus tests for expected improvements compared differences by random assignment to the treatment group. Omnibus tests for experienced improvements compared differences by random assignment to the treatment group, controlling for baseline expectation of improvement in the same domain. Omnibus tests of substance use and psychotherapy compared baseline to evaluation measures. | | | | | | | |
